# Attitudes towards and engagement in self-directed learning among paramedics in New South Wales, Australia: a cross sectional study

**DOI:** 10.1186/s12909-023-04740-0

**Published:** 2023-10-12

**Authors:** Jamie Bryant, Alison Zucca, Heidi Turon, Robert Sanson-Fisher, Alan Morrison

**Affiliations:** 1 School of Medicine and Public Health, University of Newcastle, Callaghan, NSW 2308 Australia; 2 Hunter Medical Research Institute Equity in Health and Wellbeing Program, New Lambton Heights, NSW Australia; 3 Hunter New England Population Health Unit, Hunter New England Local Health District, Wallsend, Australia; 4 NSW Ambulance Service, Rozelle, NSW Australia

**Keywords:** Paramedicine, Continuing professional development, Continuing education, Paramedic

## Abstract

**Background:**

Australian paramedics must engage in continuing professional development (CPD), including self-directed learning (SDL). This study aimed to examine paramedics’ attitudes towards training and learning activities and perceptions about what could increase engagement in self-directed CPD.

**Methods:**

A cross-sectional survey was conducted with New South Wales Ambulance paramedics. The 48-item survey examined learning attitudes, attitudes towards SDL and socio-demographic and professional characteristics.

**Results:**

Most of the 149 participants (19% consent rate) were male (74.5%) and worked full-time (96.5%). All participants agreed that paramedics should reflect on the quality of their practice (100%) and most were committed to undertaking learning to improve their skills and capability (95.2%). However, 26.3% of participants did not feel motivated to undertake learning and 58.9% did not feel supported. Paramedics reported neutral to modestly positive attitudes towards SDL. Most participants agreed they would be more likely to engage in SDL if they had access to training equipment at their station (91%) and dedicated time during work hours (90.4%).

**Conclusion:**

Paramedics are highly committed to undertaking CPD. Increased engagement may be supported by providing SDL materials at work locations and ensuring dedicated time for learning during work hours.

## Background

Continuing professional development (CPD) is a core health practitioner responsibility. It is grounded in the well-developed tradition of lifelong learning and refers to the process whereby healthcare professionals acquire new knowledge, skills, and attitudes to maintain professional standards and ensure safe, high quality and effective practice [1]. Incidental to the priority of patient safety, participation in CPD has been demonstrated to increase job satisfaction [2]. CPD is a mandatory requirement to meet professional and regulatory standards across many healthcare professions both in Australia and internationally [3]. The Paramedicine Board of Australia defines CPD as the “means by which members of the profession maintain, improve and broaden their knowledge, expertise and competence and develop the personal professional qualities required throughout their professional lives” [4].

Paramedicine entered the National Regulation and Accreditation System (NRAS) on the 1^st^ of December 2018. As a consequence, each year Australian paramedics are required to demonstrate they maintain compliance with the standards of registration determined by the Paramedicine Board of Australia. This includes engagement in CPD [5]. The Paramedicine Board of Australia CPD Registration Standard requires paramedics complete at least 30 h of CPD annually, inclusive of a minimum of 8 h that “involves a two-way flow of information and occurs with other practitioners” [4]. The overarching objective of this CPD plan is to improve patient outcomes and experiences and ensure contemporary knowledge of best practice to maximise patient safety [4, 6]. Consequently, the expectation is that CPD is not a collection of random opportunistic activities but rather an individually tailored development plan aligned to the individual’s role, practice context, scope of practice, knowledge gaps and career aspirations. In addition to their personal responsibility for meeting CPD regulatory obligations, paramedics may also be subject to credentialing standards as a condition of employment with the health service they work for. This will generally involve participation in development activities demonstrating recency and currency aligned to the scope of practice they are authorised to implement when providing care. In contrast to CPD, fulfilling credentialing requirements is generally a shared responsibility between practitioners and their employer.

CPD requirements provide opportunity for paramedics to select, as independent learners, the content and method of learning that is most suited to their learning needs and styles. This can include combinations of work-based learning (e.g., reflective practice, clinical audit), participation in formal educational courses or research, miscellaneous professional activities including providing mentorship and teaching, and self-directed learning. Self-directed learning refers to an individual taking responsibility for their own learning, with an internal motivation to develop, implement, and evaluate their approach to learning [7, 8]. Self-directed learning has been a core component of problem-based learning in medical schools for the past three decades [9]. It has considered a foundational skill for paramedics who have completed tertiary paramedic qualifications since paramedics are expected to continue and update their knowledge and understanding throughout their professional career [10]. However, it is a relatively new component of professional practice and so it is important to understand how engagement in high-quality self-directed CPD can be encouraged.

It has been suggested that in order for CPD to be effectively integrated into the paramedicine profession, four principles need to be met: (i) individuals must take personal responsibility for their own learning and development; (ii) CPD should be relevant to and feed back into practice; (iii) employers should create optimal environments for CPD; and (iv) there must be a wide and diverse range of formal and informal learning and development activities available to individuals to access [11]. These four principles are grounded in the idea that CPD should be a dynamic and inclusive process that addresses the evolving needs of both paramedics and the healthcare system. Barriers to engagement in CPD have been reported across a number of different healthcare professions [12–18], and understanding these barriers is an important precursor to developing effective and engaging CPD activities. However, Australian research on the attitudes and facilitators to participating in self-directed CPD from the perspective of paramedics is relatively limited [19]. A recent qualitative study found engagement in CPD by paramedics was influenced by modality of delivery, professional expectations, and a desire for clinical and professional improvement [19]. Paramedics were more likely to engage in CPD where there was organisational support, self-directed learning opportunities and the content of CPD was perceived as useful and relevant. They were less likely to participate when CPD had a financial or time cost and when CPD was difficult to access due to workload and rostering. However, to date, examination of the attitudes and facilitators to engagement in CPD is limited to qualitative research with small samples of paramedics. Further research is warranted to confirm and expand upon these findings and to explore additional factors that may influence CPD engagement within paramedicine.

### Aims

To better understand paramedics’ attitudes towards training and learning activities and provide insights to assist in the development of positive and effective self-directed CPD experiences, we undertook a cross-sectional survey of paramedics in NSW, Australia. We specially aimed to understand paramedics:

1. Attitudes towards training and learning activities, including engagement in SDL
2. Perceptions about what could increase their engagement in self-directed CPD.

## Material and methods

### Context

In 2019, NSW Ambulance initiated the roll out of a new self-directed training resource which allows paramedics to practice rare and complex cases, re-enact cases, and apply new skills and technology at their base work location using simulator manikins. This self-directed learning resource is referred to as the SCOPE clinical simulation resources. This research was conducted in locations that did not yet have access to the SCOPE clinical simulation resources.

### Design

A cross-sectional survey was conducted between March and October 2020 with a random sample of paramedics employed by New South Wales (NSW) Ambulance.

### Eligibility criteria

Eligible paramedics were those employed by NSW Ambulance who were currently operating at locations that did not have access to the SCOPE clinical simulation resources.

### Sampling and randomisation

Paramedics eligible to participate in the survey were identified by a representative of NSW Ambulance using staff records. A de-identified list of 3080 eligible paramedics was supplied to the research team. A sample of 800 paramedics, stratified 50:50 by metropolitan and non-metropolitan (regional) location was then randomly selected, with the number of paramedics selected from each station proportional to overall workforce size at any particular location. At least one paramedic from each location was included in the sample. The research team provided the randomly selected list of paramedics to NSW Ambulance to co-ordinate the recruitment mailout.

### Recruitment

The Director of Education for NSW Ambulance sent a Study Invitation via email to all randomly selected paramedics. This email included a copy of the Participant Information Statement, a link to the online survey and information about how to access a hard copy of the survey (if preferred). A hard copy invitation was also mailed to the station of each selected paramedic. This included a copy of the Participant Information Statement, a hardcopy survey, and a reply-paid envelope. Participants were asked to consider the study information and return their completed survey within 2 weeks if they were willing to participate. Those who completed a hard copy survey were asked to return it directly to the research team via a reply-paid envelope. The Participant Information Statement provided a telephone number to allow participants to speak to a member of the research team prior to consenting to participate if desired. Completion and return of the survey was taken as implied consent for participation. Invitations were sent in March 2020, just prior to the first wave of COVID-19 pandemic in Australia. To improve response rates, a reminder mailout, replicating the initial mail out, was sent 6 months later in September 2020. The reminder mailout explicitly instructed participants to not re-complete the survey if they had already done so. To prevent duplicate responses, survey respondents provided their first street name, high school graduation year, age, and gender, which were cross-checked in the returned surveys.

### Sample size

At the time the study was undertaken, there were approximately 4000 paramedics employed at locations across NSW. A final sample of 400 consenting participants was expected to provide sufficient data for this exploratory study. Assuming a 50% consent rate based on response rates previously achieved by NSW Ambulance in other surveys, 800 eligible paramedics were invited to participate.

### Survey

Survey items were developed in consultation with four NSW Ambulance representatives (inclusive of the Director of Education, a Duty Operations Manager, a Redesign & Innovations manager, and a paramedic educator). The survey was pilot tested with 8 NSW paramedics and refined based on feedbackbefore commencement of the study. The survey included questions in the following domains.

1. *Learning attitudes*: Twelve items assessed paramedics attitudes towards education and learning including: perceptions of personal responsibility for learning (self-reflection, actively seek training opportunities, mentorship); perceived learning benefits (clinical skills, patient outcomes); personal commitment to learning; motivation; professional and organisational support for learning; adequate training for professional registration; and professional competence. Participants were asked to respond on a four-point Likert scale—strongly disagree, disagree, agree, strongly agree.
2. *Attitudes towards engaging in self-directed learning*: Paramedics were provided with the following definition of self-directed learning “*Self-directed learning is a process in which individuals take the initiative, with or without the help of others, to determine their learning needs, formulate learning goals, identify human and material resources for learning, choose and implement appropriate learning strategies, and evaluate their learning outcomes.*” They were then presented with a five-point response scale and asked to indicate their opinion about self-directed learning with reference to two descriptors. The descriptors included: boring / stimulating; difficult / easy; a waste of time / useful; disempowering / empowering; confusing / enlightening.
3. *Strategies to support and encourage engagement with self-directed learning*: Paramedics were presented with eight strategies that might encourage engagement in self-directed learning: dedicated time to engage in self-directed learning activities during work hours; better access to resources (e.g., computer access, internet access); access to experienced paramedics in my station to support me with self-directed learning; local station manager was more supportive of self-directed learning; peers at my station were more supportive of self-directed learning; no costs associated with self-directed learning activities; no need to travel to participate in self-directed learning activities; access to self-directed training equipment and materials at my station. These strategies were derived from a literature search of common barriers to engaging in self-directed learning in the healthcare provider literature more broadly, and discussions with paramedics during survey development and pilot testing. Paramedics were asked to respond on a four-point Likert scale – strongly disagree, disagree, agree, strongly agree.
4. *Socio-demographic and professional characteristics*: Seven items explored participant age, sex, station location, paramedic role, full time or part time workload, number of years as a paramedic and paramedic training (vocational/degree).

### Analysis

Statistical analyses of quantitative data were conducted using STATA. To examine responder bias, the proportion of invited paramedics from regional (50%, *n* = 400) and metropolitan (50%, *n* = 400) locations was compared to those proportions of paramedics who responded to the survey using a z-test of proportions. Variables were summarised as frequencies and percentages for non-missing observations. Remoteness of station was determined according to Accessibility/Remoteness Index of Australia (ARIA +) using postcode.

### Ethical approval

Ethical approval was provided by the Hunter New England Human Research Ethics Committee (2019/ETH13379). All research was carried out in accordance with the Declaration of Helsinki.

## Results

### Sample characteristics

Overall, 149 surveys were completed (84 online and 65 via hardcopy), giving a response rate of 18.6%. Of these, 78 surveys were returned after the initial mailout, and a further 71 were returned after the reminder mailout. Participant demographics are provided in Table 1. The average age of participants was 40.6 years (SD = 11.5). Most participants were male (74.5%), worked full-time (96.5%), and undertook vocational training to become a paramedic (66.4%). Regional or remote based paramedics were significantly more likely to respond that metropolitan-based.” (z = 3.5, *p* < 0.001).

**Table 1:** Participant characteristics (*n* = 149)

	Participants (*n* = 149)	
	**n**	**%**
**Gender**		
Male	111	74.5
Female	38	25.5
Missing	0	-
**Paramedic Role**		
Trainee	3	2.0
Intern	4	2.7
Paramedic	105	71.4
Paramedic specialist (ALS, SCAT, SOT, Rescue, CCP)	12	8.2
Extended care paramedic	4	2.7
Intensive care paramedic	19	12.9
Missing	2	-
**Workload**		
Full time	136	96.5
Part time	5	3.5
Missing	141	-
**Training**		
Vocational	99	66.4
Undergraduate degree	50	33.5
Missing	0	-
**Station location**		
Major city	46	35.6
Regional	78	60.5
Remote	5	3.9
Missing	20	-
**Years of experience**		
≤ 5 years	51	36.4
6–10 years	20	14.3
11–15 years	20	14.3
16–20 years	27	19.3
20 years +	22	15.7
Missing	140	-

### Attitudes towards training and learning

Participant responses to questions about learning attitudes are provided in Table 2. All participants agreed that paramedics should reflect on the quality of their practice (100%; 95% CI: 100%-100%), and most reported taking responsibility for maintaining their professional competence (89.8%; 95% CI: 84.9%-94.7%), actively seek out training (85.6%; 95% CI: 79.9%-91.3%) and were committed to undertaking learning to improve their clinical skills and capability (95.2%; 95% CI: 90.4%-98.1%). However, 26.3% (95% CI: 19.1%-33.4%) of participants did not feel motivated to undertake learning to improve their clinical skills or capability, and 58.9% (95% CI: 50.5%-66.9%) did not feel supported to undertake learning to improve their clinical skills and capability. A further 21.4% (95% CI:14.7%-28.1%) did not feel supported by colleagues at their base location to undertake learning to improve their clinical skills and capability. Overall, 51.7% (95% CI:43.6%-59.9%) agreed or strongly agreed that it was difficult to complete the amount of training and learning activities to maintain professional competence, and a further 42.4% (95% CI: 34.5%-50.5%) thought it was difficult to complete the amount of training and learning activities to maintain professional registration.

**Table 2:** Attitudes towards training and learning (*n* = 147)

		Strongly disagree n (%)	Disagree n (%)	Agree n (%)	Strongly agree n (%)	Missing n
**1**	Paramedics should routinely reflect on the quality of their practice as a paramedic	0	0	50 (34)	97 (66)	0
**2**	Paramedics should take personal responsibility for maintaining their professional competence	2 (1.4)	13 (8.8)	70 (47.6)	62 (42.2)	0
**3**	Paramedics should actively seek out training opportunities to improve their professional competence	3 (2)	18 (12.3)	75 (51.4)	50 (34.3)	1
**4**	Paramedics should seek mentorship from more experienced paramedics to improve their professional competence	2 (1.4)	10 (6.9)	73 (50)	61 (41.8)	1
**5**	Training and learning activities help me to keep my skills up to date	1 (0.7)	6 (4.1)	68 (46.6)	71 (48.6)	1
**6**	Training and learning activities benefit my patients	1 (0.7)	3 (2.1)	56 (38.4)	86 (58.9)	1
**7**	I am committed to undertaking learning to improve my clinical skills and capability	1 (0.7)	6 (4.1)	80 (54.8)	59 (40.4)	1
**8**	I feel motivated to undertake learning to improve my clinical skills and capability	5 (3.5)	33 (22.8)	73 (50.3)	34 (23.5)	2
**9**	I feel supported by NSW Ambulance to undertake learning to improve my clinical skills and capability	27 (18.5)	59 (40.4)	55 (37.7)	5 (3.4)	1
**10**	I feel supported by my colleagues at my station to undertake learning to improve my clinical skills and capability	1 (0.7)	30 (20.7)	84 (57.9)	30 (20.7)	2
**11**	Completing the amount of training and learning activities required for me to maintain my professional competence is difficult	9 (6.2)	61 (42.1)	59 (40.7)	16 (11)	2
**12**	Completing the amount of training and learning activities required for me to maintain my profession registration is difficult	14 (9.6)	70 (48)	44 (30.1)	18 (12.3)	1

**Fig. 1:**
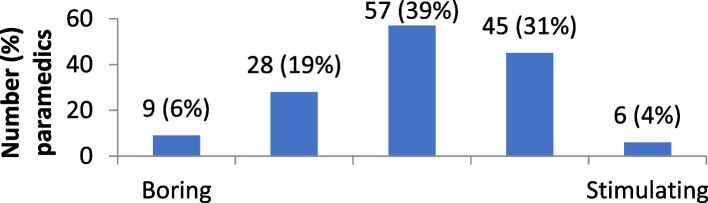
Level of interest in self-directed learning

**Fig. 2:**
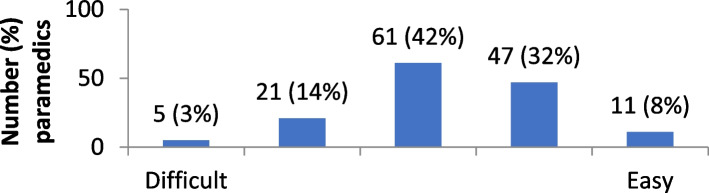
Ease of undertaking self-directed learning

**Fig. 3:**
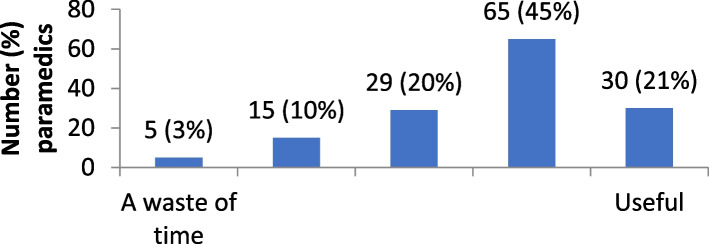
Usefulness of self-directed learning

**Fig. 4:**
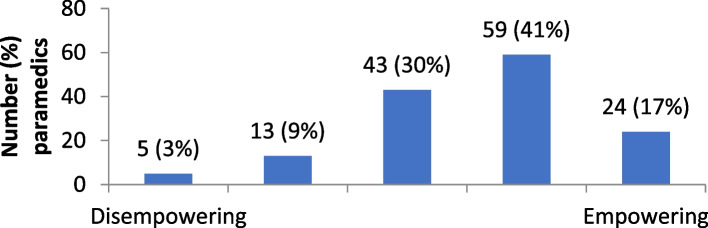
Feelings of empowerment from self-directed learning

**Fig. 5:**
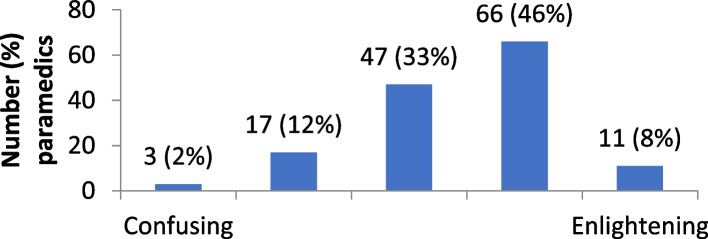
Comprehension of self-directed learning

The distribution of attitudes towards self-directed learning are provided in Figs. 1, 2, 3, 4 and 5. Overall, paramedics tended to report neutral to modestly positive attitudes towards self-directed learning. On a five-point scale, there was a modest positive skew towards self-directed learning being reported as useful (M = 3.7; SD = 1.0), empowering (M = 3.6; SD = 1.0), and enlightening (M = 3.5; SD = 0.9). Participants had more neutral attitudes about self-directed learning being interesting (M = 3.1; SD = 1.0) and easy to undertake (M = 3.2; SD = 0.9).

### Factors that would increase engagement in self-directed learning

Participant responses to questions about the barriers and enablers to engaging in self-directed learning are provided in Table 3. Most participants agreed or strongly agreed that they would be more likely to engage in self-directed learning if they had access to self-directed training equipment at their base location (91%; 95% CI: 86.4%-95.7%), dedicated learning time during work hours (90.3%; 95% CI: 85.5%-95.2%), learning activities that were free to access (80.1%; 95% CI: 73.7%-86.6%) and did not require travel (79.5%; 95% CI: 72.9%-86.0%), and they had better access to resources such as a computer or the internet (74.7%; 95% CI:67.6–81.7%). Only about half of participants agreed that they would be more likely to engage in self-directed learning if their peers and local frontline managers were more supportive (55.5% 95% CI: 47.2%-63.5%, 54.8% 95% CI: 46.7%-62.87respectively). More than two-thirds of participants (70.3%; 95% CI: 62.9%-77.8%) indicated that access to experienced paramedics in their station to support self-directed learning would result in greater engagement.

**Table 3:** Factors that would increase engagement in self-directed learning (*N* = 146)

*I would be more likely to engage in self-directed learning if:*	Strongly disagree n (%)	Disagree n (%)	Agree n (%)	Strongly agree n (%)	Missing n
**There was dedicated time to engage in self-directed learning activities during work hours**	3 (2.1)	11 (7.6)	49 (33.8)	82 (56.6)	1
**I had better access to resources (e.g., computer access, internet access)**	4 (2.7)	33 (22.6)	61 (41.8)	48 (32.9)	0
**I had access to experienced paramedics in my station to support me with self-directed learning**	3 (2.1)	39 (27.1)	60 (41.7)	42 (29.2)	1
**My local station manager was more supportive of self-directed learning**	9 (6.2)	57 (39)	61 (41.8)	19 (13)	0
**My peers at my station were more supportive of self-directed learning**	6 (4.1)	64 (43.8)	63 (43.2)	13 (8.9)	0
**There were no costs associated with self-directed learning activities**	4 (2.7)	25 (17.1)	60 (41.1)	57 (39)	0
**There was no need to travel to participate in self-directed learning activities**	5 (3.4)	25 (17.1)	62 (42.5)	54 (37)	0
**I had access to self-directed training equipment and materials at my station (e.g., simulator manikins)**	0	13 (9)	59 (40.7)	73 (50.3)	1

## Discussion

This study aimed to provide a comprehensive examination of attitudes and facilitators to engagement in self-directed CPD by paramedics in NSW, Australia, to provide insights to assist in the development of positive and effective self-directed CPD experiences. At a time when the profession of paramedicine is evolving and there is increasing professionalisation of the workforce [20], this study adds to an emerging body of knowledge attempting to understand how to best encourage engagement in high-quality ongoing CPD.

### Paramedic’s attitudes towards training and learning activities

Our findings demonstrate that most paramedics are committed to undertaking learning to improve their clinical skills and capability and feel a responsibility to do so. All paramedics thought they should reflect on the quality of their practice and most reported taking responsibility for maintaining their professional competence, actively seeking out training, and felt a commitment to improve their clinical skills and capability for the benefit of their patients. These findings reflect other qualitative research with Australian paramedics that found generally high levels of agreement about the importance of ongoing professional learning [19], and high levels of confidence in identifying and planning participation in CPD activities [21]. Paramedics also had mostly positive attitudes towards self-directed CPD, which they perceived as useful, empowering, and enlightening.

Despite general agreement about the important of CPD, just over one quarter of paramedics reported that they did not feel motivated to undertake learning to improve their clinical skills or capability. In addition, 59% of paramedics reported not feeling motivated or supported by the organisation to undertake training and learning to improve their clinical skills or capability, and one fifth reported feeling unsupported by colleagues to undertake learning to improve clinical skills and capability. Variable motivation to engage in CPD aligns with the findings of other Australian research which found that while some paramedics enjoy the process of learning, others complete CPD only because it is mandatory to maintain professional registration [21] and is part of the process of ‘jumping through the hoops’ [19]. Factors including workload, fatigue, and workplace culture issues including lack of employer support are all likely to be contributing to a reluctance to engage in educational activities beyond the minimal requirements to maintain registration [19].

### Perceptions of the factors that could increase engagement in self-directed CPD

More than 90% of participants agreed or strongly agreed that they would be more likely to engage in self-directed learning if they had access to training equipment at their work location and dedicated paid time to engage in training. A further 80% agreed that having no costs associated with self-directed learning activities would encourage uptake, and 79% said not needing to travel would increase engagement. These findings suggest that making CPD activities easily accessible and cost neutral are influential engagement factors, which aligns with both Australian and international research. Qualitative research with Australian paramedics has consistently found that financial and intangible time costs associated with participating in CPD outside of employment hours as significant barriers to participation [19, 21]. Work conducted with Danish paramedics investigated whether motivation to participate in self-directed training could be improved by increasing ease of access to training facilities by setting up a designated area at each base location with training equipment and textbooks, providing smartphone-accessible mini quizzes, erecting signs encouraging personnel to share educational stories gathered from their service, and boards with monthly themes. The study found a general increase in motivation and higher frequency of training sessions completed, with accessibility of training equipment and implementation of collegial coaching the most impactful [22], highlighting the potential contribution of dedicated paid and protected time in supporting paramedic engagement in CPD.

### Study limitations

Our study sample was primarily made up of full-time male paramedics who were vocationally educated, which does not reflect the diverse NSW ambulance workforce or their educational background. Our sample also only included paramedics from one state. As ambulance services in Australia are operated by state and territory governments with different organisational policies and structures, and NSW is unique in that it offers a direct entry/ non university pathway for paramedicine training, our findings may not be generalisable to paramedics in other states. The modest response rate and self-selection bias is a limitation of the current study and likely limits the degree to which results are representative of the views of all paramedics. Significantly more regional paramedics participated, which may reflect that regional paramedics have a stronger interest in education and training, and/or greater time to participate. Nonetheless, the response rate is consistent with that achieved in other studies with Australian paramedic populations [23, 24].

## Conclusions

Paramedics are highly committed to undertaking CPD to improve their clinical skills and capability. While most paramedics believe they have personal responsibility for maintaining their professional competence they also have an expectation that CPD should be supported and facilitated by their employer. For this cohort, engagement in high-quality ongoing professional learning may be best encouraged by optimising access to self-directed training equipment and materials at work locations and ensuring paramedics have dedicated time to engage in self-directed learning activities during work hours.

## Data Availability

The datasets used and analysed during the current study are available from the corresponding author upon reasonable request.

## References

[1] Grant J (2012). The good CPD guide: a practical guide to managed continuing professional development in medicine. Emerg Nurse.

[2] Hojat M, Kowitt B, Doria C, Gonnella JS (2010). Career satisfaction and professional accomplishments. Med Educ.

[3] Tran D, Tofade T, Thakkar N, Rouse M (2014). US and international health professions’ requirements for continuing professional development. Am J Pharm Educ.

[4] Paramedicine Board of Australia. Registration Standard: Continuing Professional Development. 2018. Available from: https://www.paramedicineboard.gov.au/Professional-standards/Registration-standards/CPD.aspx.

[5] Australian Health Practitioner Regulation Agency (AHPRA). Paramedicine Board of Australia: Professional Standards. 2018. Available from: http://www.paramedicineboard.gov.au/Professional-standards.aspx.

[6] Paramedicine Board of Australia. Guidelines: Continuing professional development. 2018. Available from: https://www.paramedicineboard.gov.au/professional-standards/registration-standards/cpd.aspx.

[7] Knowles MS (1975). Self-directed learning: a guide for learners and teachers.

[8] Gandomkar R, Sandars J (2018). Clearing the confusion about self-directed learning and self-regulated learning. Med Teach.

[9] Murad MH, Coto-Yglesias F, Varkey P, Prokop LJ, Murad AL (2010). The effectiveness of self-directed learning in health professions education: a systematic review. Med Educ.

[10] Williams B, Boyle M, Winship C, Brightwell R, Devenish S, Munro G (2013). Examination of self-directed learning readiness of paramedic undergraduates: a multi-institutional study. J Nurs Educ Pract.

[11] Martin J. The challenge of introducing continuous professional development for paramedics. Australas J Paramed. 2015;42(2). 10.33151/ajp.4.2.368.

[12] Marriott J, Duncan G, McNamara K (2007). Barriers to pharmacist participation in continuing education in Australia. Pharm Educ.

[13] Summers A (2015). Continuing professional development in australia: barriers and support. J Contin Educ Nurs.

[14] Lloyd B, Pfeiffer D, Dominish J, Heading G, Schmidt D, McCluskey A (2014). The New South Wales allied health workplace learning study: barriers and enablers to learning in the workplace. BMC Health Serv Res.

[15] Ikenwilo D, Skåtun D (2014). Perceived need and barriers to continuing professional development among doctors. Health Policy.

[16] Jeong D, Presseau J, ElChamaa R, Naumann DN, Mascaro C, Luconi F (2018). Barriers and facilitators to self-directed learning in continuing professional development for physicians in canada: a scoping review. Acad Med.

[17] Bwanga O (2020). Barriers to Continuing Professional Development (CPD) in radiography: a review of literature from Africa. Health Prof Educ.

[18] Austin Z, Gregory PAM (2017). Quality assurance and maintenance of competence assessment mechanisms in the professions: a multi-jurisdictional multi-professional review. J Med Regul.

[19] Hobbs L, Devenish S, Long D, Tippett V. Facilitators, barriers and motivators of paramedic continuing professional development. Australas J Paramed. 2021;18. 10.33151/ajp.18.857.

[20] Reed B, Cowin L, O’Meara P, Wilson I. Professionalism and professionalisation in the discipline of paramedicine. Australas J Paramed. 2019;16(0). 10.33151/ajp.16.715.

[21] Williams B, Edlington T. Attitudes towards continuing professional development: a qualitative study of Australian paramedics. Australas J Paramed. 2019;16 10.33151/ajp.16.717.

[22] Skydsgaard K (2020). Improving motivation for self-directed training in Danish EMS personnel. Int Paramed Pract.

[23] McCann TV, Savic M, Ferguson N, Cheetham A, Witt K, Emond K (2018). Recognition of, and attitudes towards, people with depression and psychosis with/without alcohol and other drug problems: results from a national survey of Australian paramedics. BMJ Open.

[24] Thyer L, Simpson P, Nugteren BV (2018). Burnout in Australian paramedics. Int Paramed Pract.

